# Therapeutic Potential of Mesenchymal Stromal Cells and MSC Conditioned Medium in Amyotrophic Lateral Sclerosis (ALS) - *In Vitro* Evidence from Primary Motor Neuron Cultures, NSC-34 Cells, Astrocytes and Microglia

**DOI:** 10.1371/journal.pone.0072926

**Published:** 2013-09-12

**Authors:** Hui Sun, Karelle Bénardais, Nancy Stanslowsky, Nadine Thau-Habermann, Niko Hensel, DongYa Huang, Peter Claus, Reinhard Dengler, Martin Stangel, Susanne Petri

**Affiliations:** 1 Department of Neurology, Hannover Medical School, Hannover, Germany; 2 Center for Systems Neuroscience, Hannover, Germany; 3 Department of Neuroanatomy, Hannover Medical School, Hannover, Germany; 4 Department of Neurology, East Hospital, Tongji University, Shanghai, China; 5 Integriertes Forschungs- und Behandlungszentrum Transplantation (IFB-Tx), Hannover, Germany; University of Lyon, France

## Abstract

Administration of mesenchymal stromal cells (MSC) improves functional outcome in the SOD1G93A mouse model of the degenerative motor neuron disorder amyotrophic lateral sclerosis (ALS) as well as in models of other neurological disorders. We have now investigated the effect of the interaction between MSC and motor neurons (derived from both non-transgenic and mutant SOD1G93A transgenic mice), NSC-34 cells and glial cells (astrocytes, microglia) (derived again from both non-transgenic and mutant SOD1G93A ALS transgenic mice) *in vitro*. In primary motor neurons, NSC-34 cells and astrocytes, MSC conditioned medium (MSC CM) attenuated staurosporine (STS) - induced apoptosis in a concentration-dependent manner. Studying MSC CM-induced expression of neurotrophic factors in astrocytes and NSC-34 cells, we found that glial cell line-derived neurotrophic factor (GDNF) and ciliary neurotrophic factor (CNTF) gene expression in astrocytes were significantly enhanced by MSC CM, with differential responses of non-transgenic and mutant astrocytes. Expression of Vascular Endothelial Growth Factor (VEGF) in NSC-34 cells was significantly upregulated upon MSC CM-treatment. MSC CM significantly reduced the expression of the cytokines TNFα and IL-6 and iNOS both in transgenic and non-transgenic astrocytes. Gene expression of the neuroprotective chemokine Fractalkine (CX3CL1) was also upregulated in mutant SOD1G93A transgenic astrocytes by MSC CM treatment. Correspondingly, MSC CM increased the respective receptor, CX3CR1, in mutant SOD1G93A transgenic microglia. Our data demonstrate that MSC modulate motor neuronal and glial response to apoptosis and inflammation. MSC therefore represent an interesting candidate for further preclinical and clinical evaluation in ALS.

## Introduction

Cellular therapy is currently being investigated as a novel therapeutic option for the treatment of neurodegenerative disorders, including Parkinson’s disease, Huntington’s disease and amyotrophic lateral sclerosis (ALS). While in some diseases such as Parkinson’s disease, cell replacement seems to be an option worth further exploration, in others such as ALS, cell therapy research rather focuses on the generation of a protective environment for degenerating neurons. This approach is supported by recent evidence that motor neuron death in ALS is non-cell autonomous and that non-neuronal cells can exert protective effects *in vitro* and *in vivo* [1], [2].

The rationale for the use of adult stem cells as a treatment for neurodegenerative diseases such as ALS comes from the notion that they are capable to provide the host tissue with growth factors or modulate the host immune system [3], [4]. Evidence from preclinical studies suggested that mesenchymal stromal cells (MSC), a subset of adult progenitor cells, can differentiate into neuronal-like cells [5]. Their neuroprotective effects, however, seem to be mainly based on anti-inflammatory and immunomodulatory activities [6]. They therefore do not require full engraftment of MSC following transplantation into the CNS, but rely on the capacity of MSC to release neuroprotective and anti-inflammatory molecules, resulting in the induction of a neuroprotective microenvironment [7], [8]. Intraspinal or intrathecal injection of human MSC delayed the onset of astrogliosis and microglia activation, motor neuron cell death, and the impairment of motor behavior in transgenic ALS mice [9], [10], [11], [12]. Other studies have similarly shown protective effects of intravenous infusion of MSC in an ALS mouse model, indicating that the most appropriate route of administration with respect to both safety and efficacy still remains to be defined [11], [13]. Bone-marrow-derived MSC have already been used in ALS patients in a clinical phase I - trial and few adverse effects have been observed which is in favour of further clinical evaluation of this approach [14], [15], [16].

Considering that motor neurons are the primary structurally and functionally impaired cells in ALS, in the present study we generated primary motor neurons derived from non-transgenic and mutant SOD1G93A transgenic ALS mice, the most commonly used and best characterized ALS animal model. In order to clarify if the beneficial effects of MSC therapy are dependent on direct cell contact or if MSC mediate neuroprotection in an indirect way, embryonic primary motor neurons were either co-cultured on MSC feeder layers or incubated with MSC conditioned medium (CM). Using staurosporine (STS) as a toxic stimulus, we tested whether MSC-co-culture or MSC CM could protect motor neurons against apoptotic stress. We further assessed the protective effects of MSC CM against STS-induced apoptosis using the motor-neuron-like cell line, NSC-34 cells, produced by fusion of motor neuron-enriched embryonic mouse spinal cord cells with mouse neuroblastoma cells, as *in vitro* motor neuron model. These cells express many of the morphological and physiological properties of primary motor neurons [17] and have the advantage to overcome the low yields and limited purity of primary motor neuron preparations. A breakthrough in ALS research was the discovery that non cell-autonomous processes due to functional dysregulation of surrounding non-neuronal cells, i.e. microglia and astrocytes, contribute to motor neuron death [1], [18]. Several studies showed that astrocytes are specific contributors to spinal motor neuron degeneration in mutant SOD1-linked ALS and that they exert toxicity on motor neurons via release of soluble factors [2], [19]. We therefore further analysed the protective effects of MSC CM pre-treatment in primary astrocyte cultures derived from either SOD1G93A or non-transgenic mice to determine whether astrocytes from mutant animals respond differently to MSC CM. As is has been extensively documented that the MAPK/Erk1/2 or PI3K/Akt signalling pathways can influence neuronal cell death and survival, we attempted to clarify whether an influence of MSC CM on these pathways is involved in the protective effects [20], [21].

MSC express a variety of cytokines and growth factors which are key mediators of central nervous system (CNS) networks [22]. Based on previous studies assessing MSC effects *in* *vitro* and *in vivo*, we therefore aimed to find out, whether expression of growth factors, cytokines and chemokines previously found to be modified by MSC and of particular interest with regard to ALS pathophysiology and therapy was influenced by MSC CM in our *in vitro* models [10], [12], [23], [24], [25]. Protective effects of growth factors, such as CNTF (ciliary neurotrophic factor), GDNF (glial cell line-derived neurotrophic factor), IGF-1 (insulin-like growth factor 1), FGF2 (basic fibroblast growth factor 2) and VEGF (vascular endothelial growth factor) [26], [27], [28], [29], [30] have been shown in rodent models of ALS. We therefore studied whether MSC CM could induce their expression in astrocytes and NSC-34 cells.

Cytokines are multifunctional proteins that have most intensively been studied regarding autoimmune diseases of the CNS such as multiple sclerosis (MS). Increasing evidence, however, suggests that inflammatory mechanisms are of major relevance in neurodegenerative diseases such as Parkinson’s [31] and Alzheimer’s disease [32]. In ALS, inflammatory mediators like tumor necrosis factor-α (TNF-α), interleukin-1 beta (IL-1ß), IL-6 and IL-10 have been suggested to play a role in the disease pathogenesis [33], [34], [35], [36], [37], [38]. The proinflammatory enzymes inducible nitric oxide synthase (iNOS) and cyclooxygenase 2 (COX2) have also been found up-regulated in human ALS and in the SOD1G93A mouse model [39], [40], [41], [42].

Based on a previous study assessing MSC effects pro-and anti- inflammatory factors *in vitro* in an astrocytic cell line, we therefore investigated whether we could reproduce MSC CM-induced modification on LPS-induced production of the cytokines TNFα, IL-6 and IL-10 and of the proinflammatory enzymes iNOS and COX2 [25] and whether there were differential effects on non-transgenic compared to SOD1G93A transgenic astrocytes.

As MSC were previously reported to modify expression of the neuroprotective chemokine fractalkine (CX3CL1) in glial cells lines [43], we also measured mRNA expression of CX3CL1 and its receptor (CX3CR1) in astrocytes and microglia.

## Materials and Methods

### Ethics Statement

All experiments were carried out in strict accordance with the internationally accepted principles in the care and use of experimental animals and were approved by the Institutional Animal Care and Research Advisory Committee at Hannover Medical School and Protection and Food Safety regional (Permit Number: AZ 07/1324).

### Animals

G93A transgenic familial ALS mice (high copy number; B6SJLTg ((SOD1-G93A)1Gur/J) [44] were obtained from the Jackson Laboratory (Bar Harbor, ME, USA). These mice over-express the human mutant SOD1 allele containing the Gly93→Ala (G93A) substitution. We maintained the transgenic G93A hemizygotes by mating transgenic males with B6SJLF1/J hybrid females. Transgenic offspring was genotyped by PCR assay of DNA obtained from tail tissue. Mice were housed under controlled conditions (12∶12 light: dark cycle) with free access to food and water. Animals of the same sex were kept in groups of up to five animals in Makrolon cages type II (UNO, Zevenaar, Netherlands). Males were kept solitary in the same cage type only when they were also used for breeding.

### Primary Motor Neuron Culture

Isolation and *in vitro* cultivation of motor neurons was performed by dissection of lumbar ventral spinal cords from individual mouse embryos (gestational age: E13/14) as previously described [44]. G93A transgenic familial ALS mice (high copy number; B6SJLTg ((SOD1-G93A)1Gur/J) [45] were obtained from the Jackson Laboratory (Bar Harbor, ME, USA). These mice over-express the human mutant SOD1 allele containing the Gly93→Ala (G93A) substitution. We maintained the transgenic G93A hemizygotes by mating transgenic males with B6SJLF1/J hybrid females. Transgenic offspring was genotyped by PCR assay of DNA obtained from the cerebellum of the embryos. After tissue dissociation, motor neurons were enriched by a p75NTR-antibody- (Abcam, Cambridge, UK) – based immunopanning technique. Culture medium (Neurobasal medium, Gibco Invitrogen, Darmstadt, Germany) with 2% horse serum, 2% B27-supplement (Invitrogen, Darmstadt, Germany); 0.5 mM glutamax-I; 5 ng/ml rHuBDNF and 5 ng/ml rHuCNTF (both from Peprotech, Hamburg, Germany) was added to the resulting pellet. Highly enriched motor neurons were seeded on glass coverslips, pre-incubated first with polyornithin (diluted 1∶1000, Sigma, Steinheim, Germany) and laminin (2.5 µg/ml, Invitrogen, Steinheim, Germany) or on a mouse MSC feeder layer at a ratio of 1∶4. The average density of motor neurons was 2.0×10^4^ cells cm^−2^. All treatment described below were performed after 7 days *in vitro* (DIV 7). The purity of primary motor neuron cultures was determined by the ratio of SMI32 positive cells to DAPI stained nuclei, resulting in approximately 80% motor neurons [46], [47].

### Microglia and Astrocyte Culture

Primary cultures were obtained from one day old non-transgenic and mutant SOD1G93A transgenic mice. Cerebra were dissected and meninges removed in HBSS/Hepes buffer. Brains were chopped with a razor blade. The brain pieces were put into 15 ml tube and were then centrifuged for 5 min at 3000 rpm at RT. For dissociation the pellet was incubated with trypsin (0.1%) at 37°C for 20–30 min, under shaking. DNase (0.001%) was added and the tubes were turned upside down several times. The tubes were centrifuged at 3500 rpm for 5 min at RT. The supernatant was discarded and the red blood cells were removed with a pasteur pipette. The remaining pellet was triturated through a flame-narrowed glass pipette until a single cell suspension was obtained. The cells were seeded in DMEM (4.5 g/L Glucose, [+] L-Glutamine, [−] Pyruvate), 10%FBS, 1% Penicillin/Streptomycin onto poly-L-lysine (diluted 1∶1000, Sigma, Steinheim, Germany) coated flasks. The next day, the culture medium was removed, cells were washed with PBS and new culture medium was added. Medium was changed every second day. On day 10–13, tissue culture flasks were closed tightly with parafilm and shaken for 30 min at 180 rpm at 37°C on an orbital shaker-incubator (Edmund Bühler, Hechingen, Germany) and plated on culture dishes (Nunc, Roskilde, Denmark). Microglial purity was more than 95% as determined by CD11b (AbD Serotec, Kidlington, UK) immunoreactivity. Medium was replaced with 12 ml DMEM/10%FBS and left in the incubator for further shaking (isolation of astrocytes). On day 14, tissue culture flasks were closed tightly with parafilm and shaken for 16 h at 170 rpm overnight at 37°C. The next day, medium with AraC (100 µM, Sigma-Aldrich, Steinheim, Germany) was added to each flask. The flasks were incubated at 37°C for 72 h. After washing with PBS, cells were trypsinised, counted and plated at a density of 3×10^4^ cells cm^−2^ for RNA extraction. For immunocytochemistry, cells were grown on poly-L-Lysine coated coverslips in 24 well plates at a density of 1×10^4^ cells cm^−2^.

### Staurosporine-induced Toxicity

Highly enriched motor neurons derived from either non-transgenic or mutant SOD1G93A transgenic ALS mice (non-tg MN/G93A MN) were seeded on glass coverslips either pre-incubated with polyornithin (diluted 1∶1000, Sigma, Steinheim, Germany) and laminin (2.5 µg/ml, Invitrogen, Steinheim, Germany) or covered with a mouse MSC feeder layer. The ratio of MSC to motor neurons was 1∶4. The average density of motor neurons was 2.0×10^4^ cells cm^−2^. NSC-34 cells and astrocytes were both seeded on poly-L-lysine (diluted 1∶1000, Sigma, Steinheim, Germany) coated glass coverslips. The average density of NSC-34 cells and astrocytes was 2.0×10^4^ cells per cm^2^.

After 7 DIV, monocultures of non-transgenic motor neurons as well as motor neuron-MSC cocultures were incubated for 24 h with different concentrations of staurosporine (STS) (0.1, 0.2, and 0.5 µM, respectively) to test for concentration dependent neurotoxic effects. NSC-34 cells and astrocytes were incubated for 24 h with different concentrations of STS (NSC-34 cells: 0.03, 0.1, 0.2, 0.3, 0.5 and 1 µM; Astrocytes: 0.1, 0.2, 0.5 and 1 µM, respectively) to test for concentration dependent neurotoxic effects using the MTT assay.

### Conditioned Medium and Cell Treatments

MSC derived from bone marrow of C57BL/6 mice were obtained from Gibco (Karlsruhe, Germany). Cells were cultured in D-MEM/F-12 medium with GlutaMAX™-I (Gibco, Karlsruhe, Germany) supplemented with 10% MSC-Qualified fetal bovine serum (FBS, Gibco, Karlsruhe, Germany) and 1% Penicillin/Streptomycin (Sigma–Aldrich, Steinheim, Germany). MSC were seeded at a density of 3.0×10^3^ per cm^2^. After rinsing with PBS, Neurobasal medium supplemented with 1% B27, 1% Penicillin/Streptomycin, 0.5 mM L-glutamine, and 2% FBS was added. After 24 h, supernatants from MSC cultures were collected, and centrifuged at 3000 rpm for 5 min to remove remaining cells. Debris was removed by rinsing the supernatant through a 0.22 µm filter and designated as conditioned medium (CM) for primary motor neurons. Aliquots of CM were frozen at −80°C. In order to get conditioned medium for NSC-34 cells, DMEM (low glucose 1 g/L; GmbH, Aidenbach, Germany) was used instead of Neurobasal medium. Conditioned medium for astrocytes was obtained by growing MSC in DMEM (4.5 g/L Glucose, [+] L-Glutamine, [−] Pyruvate), 10% FBS, 1% Penicillin/Streptomycin.

**Figure 1:**
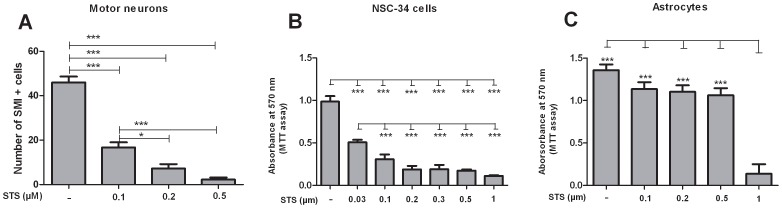
Assessment of staurosporine-induced toxicity in motor neurons, NSC-34 cells and astrocytes. Dose dependent neurotoxic effect of STS on non-transgenic motor neurons cultured in monoculture as quantified by immunocytochemistry at DIV 7 (n = 8, Fig. 1A). Significant toxic effect of different concentrations of STS on viability of NSC-34 cells (Fig. 1B) and non-transgenic astrocytes (Fig. 1C) as detected by MTT assay. Values represent means ± SEM, ***p<0.001, *p<0.05. One-way ANOVA with Bonferroni post-test.

CM was added at different concentrations ranging from 20% to 100% (V/V) in different culture medium either 4 h before induction of apoptosis or simultaneously together with the proapoptotic stimulus. Apoptosis was induced by staurosporine (STS), based on dose-finding experiments in each cell type (Fig.1) (0.1 µM for primary motor neurons; 0.03 µM for NSC-34 cells; 1 µM for astrocytes; Sigma, Steinheim, Germany) for 24 h.

Microglia were co-cultured for 24 hours in the presence or absence of MSC at 1∶1 ratio (3×10^5^ cells) in a six - well plate in a transwell system (BD FALCON Cell Culture Inserts, BD Biosciences, San Diego, CA).

### Immunocytochemistry for Assessment of Survival of Motor Neurons, NSC-34 Cells and Astrocytes

Cells were fixed with 4% paraformaldehyde (PFA). Motor neurons were stained over night with an antibody against SMI32 (1∶1000; monoclonal, Cambridge, UK) which is specific for motor neurons derived from embryonic spinal cord. MSC were identified with CD44 (1∶50; monoclonal, BD Biosciences, Heidelberg, Germany). NSC-34 cells were stained over night with an antibody against ß-III tubulin (1∶140, Abcam, Cambridge, UK) and astrocytes with an antibody against glial fibrillary acidic protein (GFAP; 1∶300; Sigma, polyclonal, Dako Cytomation, Glostrup, Denmark). Appropriate Alexa Fluor 555 or Alexa Fluor 488 coupled secondary goat anti-mouse or goat anti-rabbit antibodies (Invitrogen, Darmstadt, Germany) were applied for 45 min at RT. Cell nuclei were stained with 4′, 6-diamidino-2-phenylindole (DAPI; Invitrogen, Darmstadt, Germany). Motor neuron and astrocyte survival was quantified by cell counting in 5 visual fields of each coverslip in a total of eight different preparations for both non-transgenic and transgenic cells, as previously described [46], [47].

### MTT Cell Viability Assay

Cell viability of NSC-34 cells and astrocytes after STS treatment was assessed via the MTT (3-(4, 5-dimethylthiazol-2-yl)-2, 5-diphenylte-trazolium bromide) assay (Sigma, Steinheim, Germany), which measures the ability of cells to reduce MTT to formazan. After acid isopropanol extraction, formazan absorbance was quantified at 570 nm with a reference wavelength of 630 nm (Tecan spectrophotometer, Salzburg, Austria).

### Blocking Experiment

The PI3-K inhibitor Ly294002 and the MEK-1 inhibitor PD98059 (both from Biotrend, Cologne, Germany) were applied to both the NSC-34 cells and astroglial cultures at a final concentration of 10 µM 1 h prior to incubation with CM, hence 5 h prior to induction of apoptosis. The inhibitors were left in the medium during the whole pre-incubation period and were present after induction of apoptosis until the evaluation of apoptotic cells 24 h later.

### Quantitative Real-time Polymerase Chain Reaction (qRT-PCR)

Astrocytes and NSC-34 cells were harvested and total RNA was extracted according to the manufacture’s protocol using RNeasy Micro Kit (Qiagen GmbH, Düsseldorf, Germany). Reverse transcription of 1500 ng total RNA per reaction was carried out using oligo-dT primer and Superscript II reverse transcriptase (Invitrogen, Darmstadt, Germany). The genetic expression of growth factors and cytokines were quantified using the TaqMan method with the following assays synthesized by Lifetech (Life Technologies; Applied Biosystems): GDNF (Mm00599849_m1), BDNF (Mm01334042_m1), CNTF (Mm00446373_m1), FGF-2 (Mm00433287_m1), VEGF (Mm00437304_m1), NGF (Mm00443039_m1), IGF (Mm00439560_m1), TNFα (Mm99999068_m1), IL-6 (Mm00446190_m1), iNOS (Mm00440502_m1), COX2 (Mm03294838_g1) and IL-10 (Mm00439614_m1), CX3CL1 (Mm00436454_m1), CX3CR1 (Mm00438354_m1). Hprt1 (Mm 00446968_m1) was used as reference gene for astrocytes. Gapdh (Mm99999915_g1) was used as reference gene for NSC-34 cells. Corresponding to mRNA, cDNA was used at a concentration of 25 ng/µL. qRT-PCR was performed with cDNA from 50 ng total RNA and TaqMan®Fast Universal Master Mix (Applied Biosystems) on a StepOnePlus instrument (Applied Biosystems) under the following standard conditions: 95°C for 20 s, followed by 40 cycles of 95°C for 1 s and 60°C for 20 s. The relative gene expression was calculated via the comparative Ct method as previously described by K. Livak (Applied Biosystems User Bulletin #2, 2001). Ct values were normalized to Hrtp1 and used to calculate the relative gene expression using the 2^−ΔΔCt^ method [29].

### Statistical Analysis

All results were expressed as mean ± SEM. GraphPad Prism 3.0 software was used for statistical evaluation. Comparisons between different conditions were performed using one-way ANOVA and two-way ANOVA with Bonferroni post-test.

## Results

### Staurosporine (STS) Induces Apoptosis in Primary Motor Neurons, NSC-34 Cells and Astrocytes

STS induced cytotoxicity was used to model *in vitro* motor neuron damage, because apoptotic motor neuron death is of major relevance in ALS. To induce apoptosis, DIV 7 non-transgenic motor neuron monocultures were exposed to STS in concentrations ranging from 0.1 to 0.5 µM for 24 h (Fig. 1A). A significant reduction in motor neuron number was observed following incubation with 0.1 and 0.5 µM STS and significant dose-dependency of STS-toxicity was observed between 0.1 and 0.5 µM, and 0.1 µM and 0.2 µM. In light of these results, 0.1 µM STS was chosen for further experiments with primary motor neurons.

In NSC-34 cells, a significant decrease in cell viability was observed following incubation with 0.03 µM, 0.1 µM, 0.2 µM, 0.3 µM, 0.5 µM and 1 µM STS for 24 h. No dose dependent effect of STS toxicity on NSC-34 cells could be observed. 0.03 µM STS was sufficient to reduce cell viability by about 50% (Fig. 1B). Thus, 0.03 µM STS was chosen for the further experiments with NSC-34 cells.

In astrocyte monocultures, significant reduction of cell viability was only observed following incubation with 1 µM STS, as determined by MTT assay. Therefore, 1 µM STS was used for further experiments with astrocyte cultures (Fig. 1C).

### MSC-conditioned Medium (CM) Protects Primary Motor Neurons, NSC-34 Cells and Astrocytes against Staurosporine Toxicity in a Concentration Dependent Manner

**Figure 2:**
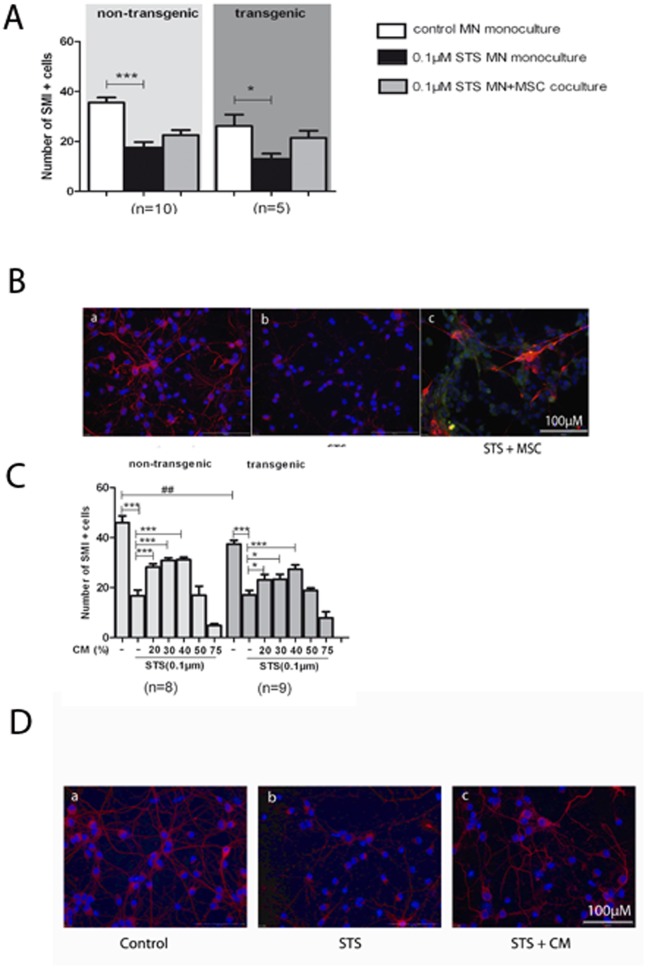
Protective effects of MSC-co-culture and of MSC conditioned medium (MSC CM) against STS toxicity in motor neurons. A: The co-cultivation of motor neurons on MSC showed a slight but not significant protective effect against STS toxicity, with greater STS-sensitivity of SOD1G93A motor neurons. Values represent means ± SEM, ***p<0.001, *p<0.05, two-way ANOVA with Bonferroni post-test. B: Photographs show representative motor neuron cultures at DIV 7 (B). a: SOD1G93A transgenic motor neuron monoculture without any treatment. b: Reduction of SMI 32 positive motor neurons following exposure to 0.1 µM STS. c: Increase in motor neuron survival due to co-cultivation on MSC. Motor neurons were stained by an antibody against SMI 32 (red). MSC were immunopositive for CD44 (green). Stained nuclei of cultured cells appear in blue (DAPI). Scale bar 100 µm. C: STS-induced apoptosis was attenuated best by 40% dilution of MSC CM, whereas at concentrations higher than 50% CM did not mediate neuroprotection. SOD1G93A motor neurons were significantly more sensitive to STS than non-transgenic motor neurons. Values represent means ± SEM, ***p<0.001, **/^##^ *p*<0.01, *p<0.05, two-way ANOVA with Bonferroni post-test. D: Immunostainings revealed the apoptotic morphology of motor neurons after STS treatment (b) and the protective effect of MSC CM (c).

Our study aimed to further investigate the cellular interactions between MSC and motor neurons *in vitro*. In order to clarify whether the protective effects of MSC are attributable to direct cell interaction or rather to MSC-released factors which mediate neuroprotection in an indirect manner, mouse primary non-transgenic and mutant SOD1G93A transgenic motor neurons were either cultured in co-culture with MSC or incubated with MSC conditioned medium (MSC CM) prior to the STS-induced apoptotic insult. In motor neuron-MSC co-cultures, a not-significant protective effect of MSC against STS-induced toxicity was observed in both non-transgenic and transgenic motor neurons (Fig. 2A). STS-sensitivity of SOD1G93A transgenic motor neurons was significantly greater than of non-transgenic motor neurons (Fig. 2C). As shown in Fig. 2C, when the culture medium was substituted with MSC CM to a quantity of 40%, STS-induced apoptosis was significantly attenuated. In non-transgenic motor neuron cultures the maximum protective effect was already reached with 20% CM, whereas in transgenic cells 40% CM resulted in the highest percentage of surviving motor neurons. Interestingly, CM concentrations higher than 40% did not result in any protective effect (Fig. 2C). Protective effects of MSC CM were only observed when it was added 4 h before STS while administration of STS and MSC CM at the same time point did not result in significant neuroprotection (not shown).

**Figure 3:**
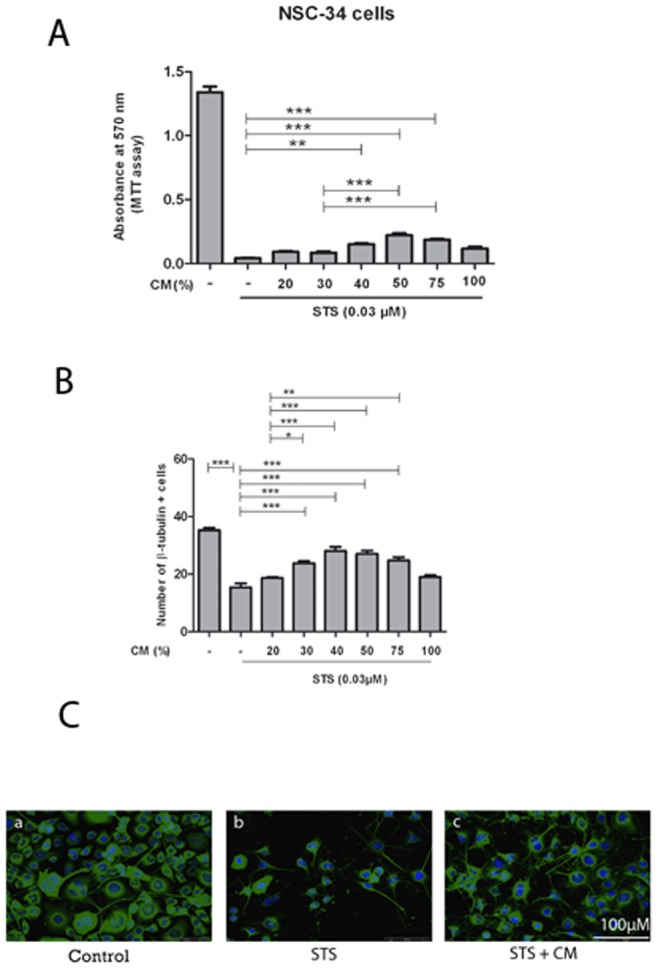
Protective effects of MSC CM against STS toxicity in NSC-34 cells. NSC-34 cells were incubated with DMEM conditioned by MSC (CM) starting 4 h before exposure to STS (0.03 µM). A: STS-induced apoptosis was attenuated best by a 50% dilution of CM, as shown by MTT assay. B: Quantification of cell death by immunocytochemical analysis similarly revealed most neuroprotection at a 10–50% dilution of MSC CM. Values represent means ± SEM, ***p<0.001, **p<0.01, *p<0.05, one-way ANOVA with Bonferroni post-test. C: Immunostainings showed a reduction of cell number due to induction of apoptosis by STS, as well as the protective effect of MSC CM. a: NSC-34 cell monoculture without any treatment. b: Reduction of ß-III tubulin positive NSC-34 cells following exposure to 0.03 µM STS. c: Increase in NSC-34 cell survival due to CM treatment. NSC-34 cells were stained by an antibody against ß-III tubulin (green). Stained nuclei of cultured cells appear in blue (DAPI). Scale bar 100 µm.

**Figure 4:**
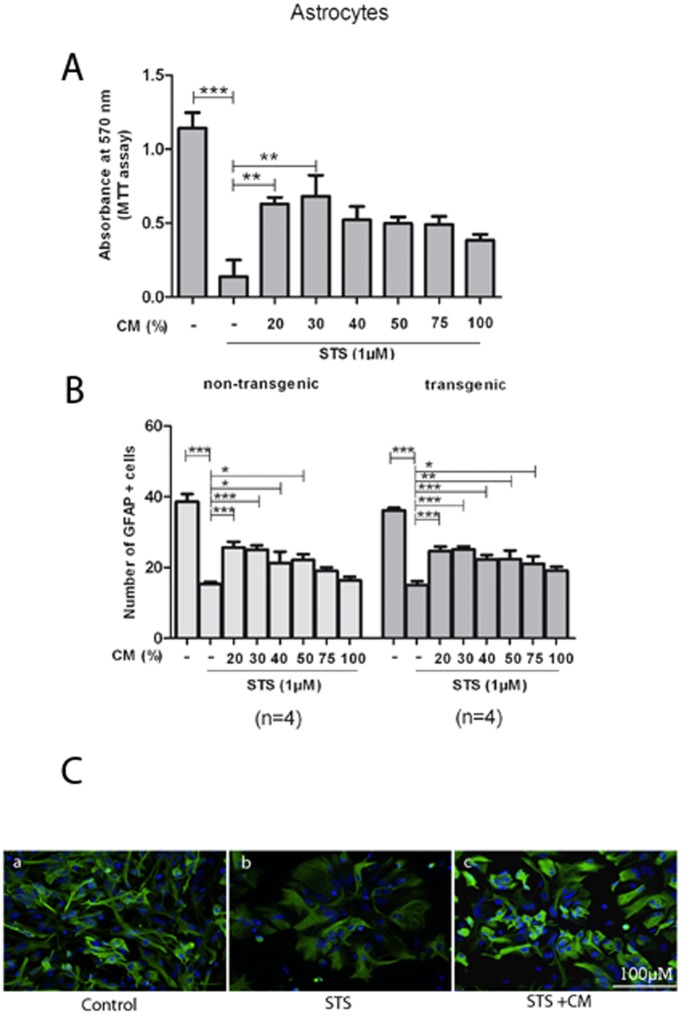
Influence of MSC CM on astrocytes under STS treatment. Astrocyte cultures were examined at high magnification by fluorescence microscopy after GFAP staining. Cultures were incubated for 48 h in conditioned medium, 1 µM STS was added during the last 24 h. A: MTT assay showed significantly increased survival of non-transgenic astrocytes in presence of 20% and 30% CM. B: Immunocytochemical quantification of astrocyte survival showed most efficient attenuation of STS-induced apoptosis with a 30% dilution of CM, whereas concentrations higher than 50% CM did not mediate significant protective effects. STS-sensitivity of non-transgenic and SOD1G93A transgenic astrocytes did not significantly differ. Values represent means ± SEM, ***p<0.001, **p<0.01, *p<0.05, two-way ANOVA with Bonferroni post-test (Fig. 4B); one-way ANOVA with Bonferroni post-test (Fig. 4A). C: Immunostainings revealed STS-induced apoptosis of astrocytes (a as compared to b) and a protective effect of MSC CM (c).

Similar neuroprotective effects were observed with MSC CM in NSC-34 cells (Fig. 3) and astrocytes (Fig. 4). In NSC-34 cells, MSC CM significantly reduced STS-induced apoptosis with a maximum protective effect at 40–50% CM (Fig. 3). STS-induced apoptosis in astrocytes was most attenuated by 30% CM (Fig. 4). These results demonstrate that MSC CM protects both SOD1G93A and non-transgenic primary motor neurons, NSC-34 cells and astrocytes against STS-induced apoptosis in a concentration dependent manner.

### Potential Involvement of *MAPK/Erk1/2 and PI3K/Akt* in Neuroprotective Effects of MSC CM in NSC-34 Cells and Astrocytes

**Figure 5:**
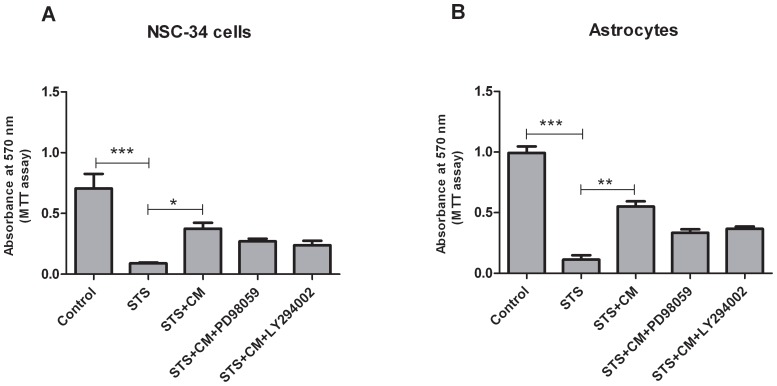
Involvement of the MAPK/Erk1/2 and PI3-K/Akt pathways in MSC CM-mediated protection. 10 µM MAPK/Erk1/2 inhibitor PD98059 and 10 µM PI3-K/Akt inhibitor LY294002 were applied to both NSC-34 cells (A) and astrocytes (B) 1 h before CM co-incubation and 5 h before induction of apoptosis by STS (0.03 µM for NSC-34 cells, 1 µM for astrocytes). This pre-incubation with PD98059 and LY294002 lowered the protective effect of CM against STS-induced apoptosis resulting in a no longer significant increase in cell survival as compared to STS-exposure. Values represent means ± SEM, ***p<0.001, **p<0.01, *p<0.05, one-way ANOVA with Bonferroni post-test.

The MAPK/Erk1/2 and PI3-K/Akt pathways are important signalling cascades that can mediate differentiation, proliferation, growth and survival of neurons and astrocytes [48]. In order to evaluate a potential functional role of these pathways in the anti-apoptotic effect of MSC CM, they were blocked by specific inhibitors. Pre-incubation with PD98059, a specific inhibitor of MEK that blocks the activation of the MAPK/Erk1/2 pathway, abolished the significant MSC CM-mediated neuroprotection after STS induced apoptosis in both NSC-34 cells and astroglial cultures (Fig. 5). Likewise, the protective effect of MSC CM against STS toxicity was reduced and no longer significant after pre-incubation with LY294002 (Fig. 5). Altogether, these findings point towards a functional involvement of these survival signalling pathways in the protective effect of MSC CM towards apoptotic cell death in NSC-34 cells and astrocytes.

### GDNF and CNTF Expression is Upregulated by MSC CM in Astrocytes, VEGF Expression is Upregulated by MSC CM in NSC-34 Cells

**Figure 6:**
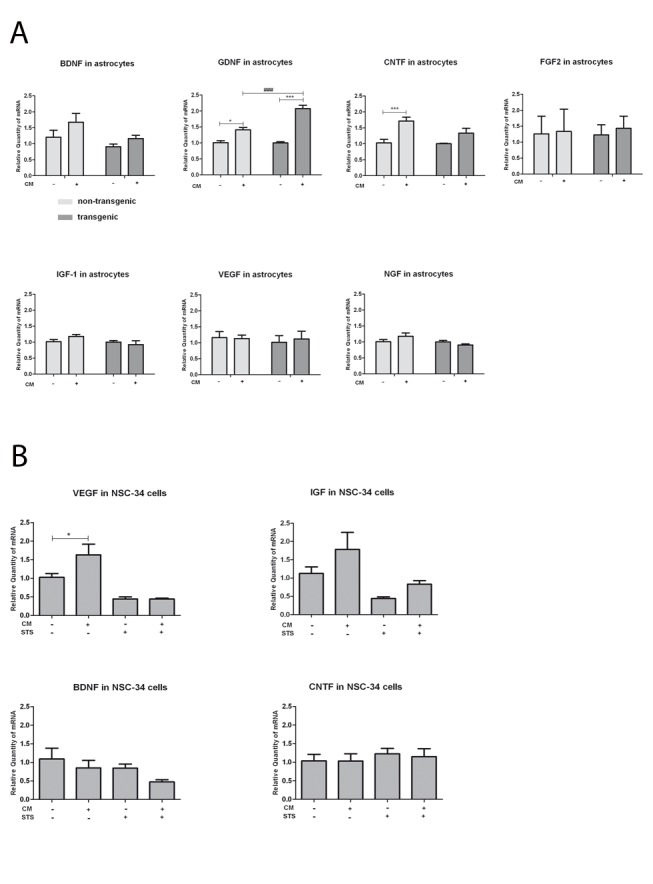
MSC CM induces expression of GDNF and CNTF in astrocytes, and induces expression of VEGF in NSC-34 cells. The influence of MSC CM on growth factor (BDNF, GDNF, CNTF, FGF2, IGF-1, VEGF and NGF) expression of astrocytes (A) and NSC-34 cells (B) was measured by qRT-PCR. mRNA expression of GDNF in both non-transgenic and SOD1G93A transgenic astrocytes was significantly up-regulated by MSC CM. The GDNF mRNA level upon incubation with 100% CM was significantly higher in SOD1G93A transgenic astrocytes compared to non-transgenic ones. CNTF mRNA expression was significantly up-regulated in non-transgenic astrocytes and slightly but not significantly up-regulated in SOD1G93A transgenic astrocytes. BDNF, FGF2, IGF-1, VEGF and NGF expression was not significantly modified (A). The mRNA level of VEGF was significantly up-regulated in NSC-34 cells under MSC CM treatment (B) while IGF, BDNF and CNTF remained unaltered and NGF, FGF-2 and GDNF were not detectable. Values represent means ± SEM, ^###^/***p<0.001, ^##^ *p*<0.01, *p<0.05, two-way and one-way ANOVA with Bonferroni post-test.

To determine if co-incubation with MSC CM could result in self-mediated protection and survival of astrocytes and NSC-34 cells as well as in neuroprotection due to increased astrocytic and NSC-34 cells release of neurotrophic factors, gene expression of BDNF, VEGF, IGF, NGF, FGF2, GDNF and CNTF was measured in both non-transgenic and mutant SOD1G93A astrocytes and NSC-34 cells. The presence of MSC CM significantly upregulated mRNA levels of GDNF in both non-transgenic and SOD1G93A transgenic astrocytes but the increase was significantly higher in mutant SOD1G93A astrocytes in comparison to non-transgenic astrocytes (Fig. 6A). CNTF mRNA expression was significantly upregulated upon MSC CM exposure in non-transgenic astrocytes while this effect was not significant in SOD1G93A astrocytes (Fig. 6A). VEGF mRNA expression was significantly upregulated upon MSC CM exposure in NSC-34 cells (Fig. 6B). Quantification of gene expression of BDNF, CNTF and IGF did not result in significant differences (Fig. 6B). This indicates that protective effects of both non-transgenic and SOD1G93A transgenic astrocytes may be enhanced by MSC CM via increase in growth factor secretion and that MSC CM can also stimulate neuronal growth factor expression.

### MSC CM Influences Astrocytic Gene Expression

**Figure 7:**
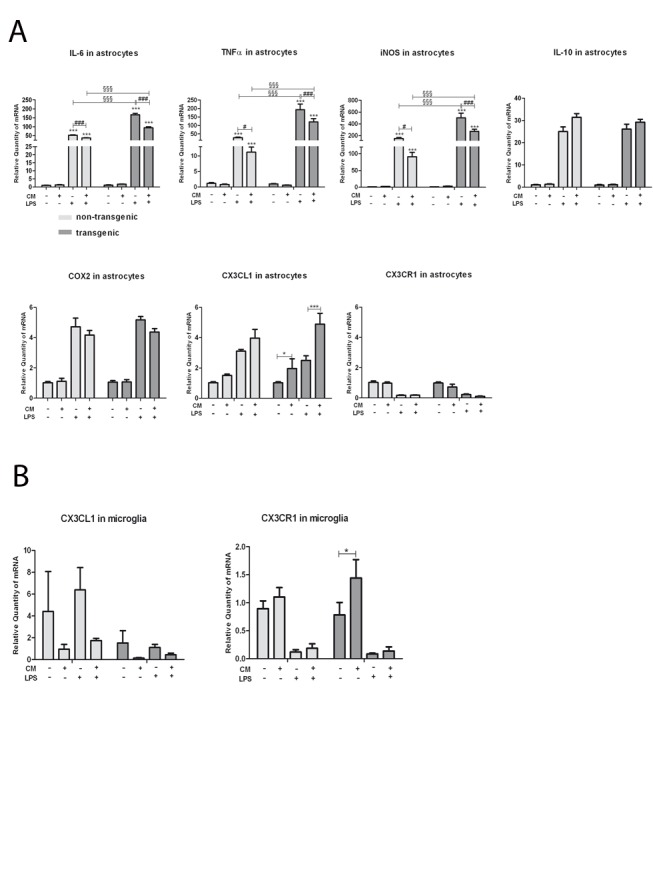
Influence of MSC CM on astrocytic and microglial gene expression. The relative mRNA expression of the pro-inflammatory cytokines TNFα and IL-6, the anti-inflammatory cytokine IL-10, the pro-inflammatory enzymes iNOS and COX2, and the neuroprotective chemokine CX3CL1 and its receptor CX3CR1 were examined in non-transgenic and SOD1G93A transgenic astrocyte cultures incubated for 28 h in 100% MSC CM or regular medium without CM and with or without LPS stimulation (500 ng/mL, added during the last 4 h) (A). The relative mRNA expression of the corresponding receptor CX3CR1 was analysed in non-transgenic and SOD1G93A transgenic microglia cultures co-cultured with or without MSC in a transwell system (B). LPS-induced upregulation of the pro-inflammatory cytokines IL-6 and TNFα and of the pro-inflammatory enzyme iNOS was significantly higher in transgenic than in non-transgenic astrocytes and was significantly attenuated by MSC CM (A). MSC CM significantly upregulated CX3CL1 expression in SOD1G93A transgenic astrocytes, while the MSC CM induced increase in CX3CL1 in non-transgenic astrocytes was not statistically significant. MSC significantly increased the expression of CX3CR1 in transgenic microglial cells, and slightly but not significantly upregulated it also in non-transgenic ones (B). Values represent means ± SEM, §§§/^###^/***p<0.001, ^#^/*p<0.05, two-way ANOVA with Bonferroni post-test.

Using lipopolysaccharide (LPS)-induced activation of astrocytes, we wanted to find out if functional adaptations to inflammation were influenced by MSC CM. The pro-inflammatory cytokines TNFα and IL-6, as well as the pro-inflammatory enzyme iNOS are typically upregulated under inflammatory conditions and take part in a pro-inflammatory immune response [49], [50]. LPS-induced upregulation of TNFα, IL-6 and iNOS was significantly higher in SOD1G93A transgenic than in non-transgenic astrocytes. In cells that had been exposed to MSC CM, the LPS-induced expression of TNFα, IL-6 and iNOS was significantly decreased in both non-transgenic and SOD1G93A transgenic astrocytes, but the decrease was significantly greater in SOD1G93A transgenic astrocytes compared to non-transgenic ones, while expression of COX-2 and IL-10 was not significantly modified (Fig. 7A).

### MSC Increase Expression of Neuroprotective Molecules in Astrocytes and Microglia

We analyzed the effect of MSC on mRNA expression of the neuroprotective chemokine CX3CL1 and its receptor CX3CR1 in both astrocytes and microglia. MSC CM significantly upregulated CX3CL1 mRNA in SOD1G93A transgenic astrocytes after LPS-treatment (Fig. 7A). The mRNA expression of CX3CR1 was significantly upregulated by co-culture with MSC in SOD1G93A transgenic microglia, and slightly but not significantly upregulated in non-transgenic microglia (Fig. 7B).

## Discussion

The present study demonstrates that MSC have protective effects on primary motor neurons, NSC-34 cells and astrocytes against STS-induced apoptosis. In comparison MSC CM was even more efficient than co-culture of motor neurons together with MSC in a ratio of 1∶4, indicating that the effects are mediated in a paracrine manner via release of protective factors by MSC, that exert their effects across cell type barriers, which is typical for growth factors and cytokines. Use of MSC CM probably resulted in higher concentrations of MSC-secreted factors than the co-culture of MSC and motor neurons. In microglial cells, on the other hand, co-culture with MSC was sufficient to induce increased expression of CX3CR1. Further studies should assess whether a higher MSC to motor neuron ratio may result in more significant motor neuron protection. For translation into clinical applications, one must further consider that in our study the induction of apoptosis via exposure to staurosporine rather represents a situation of acute toxicity for which MSC CM with higher concentrations of secreted factors could be more efficient. In the chronic neurodegenerative condition of ALS, on the other hand, long term protective effects, which are more likely to arise from transplanted cells, might be required. Ultimately, *in vivo* studies in animal models are necessary to determine in which concentration and frequency MSC should be transplanted to yield maximum protective effects as well as which mode of administration is most efficient.

While protective effects of MSC CM had already been demonstrated in rodent hippocampal neurons [7] and astrocytes [48], in the present study, we showed for the first time that MSC CM has protective effects on primary non-transgenic and SOD1G93A transgenic motor neurons (with greater sensitivity but similar rescue effects for the latter ones), on NSC-34 cells, a cell line with motor neuron characteristics, and on primary astrocytes derived from either non-transgenic or SOD1G93A mice. It is important to note that MSC CM exerted protection in a concentration-dependent manner, depending on the cell type investigated. Significant protective effects were observed at dilutions of 20–75%. At concentrations higher than 75%, however, the protective effect of MSC CM was diminished in all investigated cell types. This observation is in accord with one previous study where a similar concentration dependency of the protective effects of MSC CM was observed in rat hippocampal neurons [7]. As already discussed there, this could render clinical application of MSC in neurological disorders problematic, given that too high concentrations of MSC may result in accelerated neuronal apoptosis and increased tissue damage [7]. First clinical studies have however shown that transplantation of MSC in patients with ALS is a clinically feasible and relatively safe procedure and induces immediate immunomodulatory effects [15], [51].

To gain further insights into the mechanisms underlying the protective effects of MSC CM on motor neurons, NSC-34 cells and astrocytes against STS induced apoptosis we examined involvement of both the MAPK/Erk1/2 and PI3K/Akt pathways. They represent the most prominent survival signalling cascades that can be stimulated in neurons and astrocytes by a wide variety of growth factors or cytokines [48], [52]. Whether these pathways were involved in the protective effects of MSC transplantation in the previous *in vivo* studies in ALS mice [9], [11] has not yet been studied. Here, we demonstrated that activation of the MAPK/Erk1/2 and PI3K/Akt pathways is involved in the protective effects of MSC CM in motor neurons and astrocytes as CM-mediated protection against STS - induced apoptosis was attenuated by the MEK-1 inhibitor PD98059 and the inhibitor of PI3-K LY294002 in NSC-34 cells and astrocytes. This observation is in line with previous studies which showed that MSC CM activated phosphorylation of MAPK/Erk and/or PI3K/Akt in primary rat DRG neurons [7], [8], [48].

Our study provides the first experimental evidence for an influence of MSC CM not only on non-transgenic but also on mutant SOD1G93A transgenic astrocytes. Gene expression of neurotrophic factors was differentially regulated depending on the genotype of the astrocytes: while MSC CM induced GDNF expression was significantly higher in SOD1G93A transgenic astrocytes, CNTF mRNA expression was significantly increased upon MSC CM incubation only in non-transgenic astrocytes. This highlights the notion that disease-specific differences and interactions in neurotrophic factor expression profiles may be relevant in ALS pathogenesis [29]. We also observed that exposure of NSC-34 cells to MSC CM caused significant induction of VEGF expression which has also been implied in ALS pathophysiology and neuroprotection [53], [54]. These data further suggest that MSC induced protection of mutant transgenic astrocytes and motor neurons involve regulation of neurotrophic factor production and subsequent effects on both astrocytes themselves and motor neurons. This is in accord with a previous study which found higher GDNF immunoreactivity in the spinal cord of the motor neuron disease mouse model after intraspinal MSC injection [55]. Beneficial effects of intra-muscular injection of MSC that were genetically modified to release increased levels of GDNF have also been reported in a rat model of ALS [55], [56]. One study showed that daily applications of CNTF protected fast twitch and fast fatigable (FF) axons from synaptic vesicle loss and increased axonal resistance in ALS mice [57]. These growth factors could also be involved in activation of MAPK/Erk1/2 and PI3K/Akt survival signalling pathways. It has been well established that GDNF promotes differentiation and survival in neurons by acting on receptor tyrosine kinases (Trk), and downstream activation of PI3-K/Akt and MAPK pathways [58], [59]. One study also showed that the effect of CNTF on the viability of muscle progenitor cells (MPCs) was mediated via the PI3-Akt pathway [60].VEGF can protect spinal motor neurons via blocking p38MAPK [61].

Besides the influence on neurotrophic factor gene expression levels, we also analysed the effects of MSC CM on astrocytes, pre-incubated with MSC CM before induction of inflammation by LPS. mRNA expression of the cytokines TNF alpha and IL-6 as well as of iNOS was pronouncedly induced by LPS as expected, with more increased reactivity of SOD1G93A transgenic astrocytes, and significantly down regulated by pre-incubation with MSC CM. Several studies have similarly described an altered expression of inflammatory molecules upon MSC treatment [25], [62], [63]. One must therefore conclude that this down regulation of pro-inflammatory factors is mediated via factors released by MSC. This modulation of inflammatory cytokines and enzymes certainly contributes to the therapeutic benefit of MSC administration which has been demonstrated *in vivo* in models of ALS, inflammatory and autoimmune disease [6], [9], [11], [62], [64], [65].

The CX3CL1– CX3CR1 axis may play an important role in immunoregulation in several neurodegenerative diseases such as PD, AD and ALS [66], [67], [68], [69]. In our study, first, we observed that MSC CM increased astrocytic CX3CL1 expression upon LPS treatment. Second, we demonstrated that MSC can induce the expression of the corresponding receptor CX3CR1 in SOD1G93A transgenic microglia. This was in accord with a recent study which showed that MSC exert beneficial effects on activated microglial cells of the N9 cell line in altering their phenotype and reactivity towards a more neuroprotective phenotype via release of the chemokine CX3CL1 and interaction with microglial CX3CL1 receptors (CX3CR1) [43]. Our results indicate that MSC could provide neuroprotective effects in ALS not only by direct release of CX3CL1 but also by stimulation of astrocytic CX3CL1 secretion and microglial CX3CR1 expression. Protective effects of exogenous CX3CL1 administration has largely been shown in animal models of neuroinflammation [70], [71] but plasma levels of soluble CX3CL1 were also positively correlated with disease severity and progression in human PD patients, indicating that CX3CL1 also plays a role in chronic neurodegeneration [72]. Another study showed that enhancing CX3CR1 expression protects against microglial neurotoxicity [69].

In summary, our data indicate that MSC CM exerts a protective role against *in vitro* induced apoptosis in different cell types (primary motor neurons, NSC-34 cells and astrocytes) and maintains its protective potential in motor neurons and astrocytes carrying the ALS-causing SOD1G93A mutation. This function may involve the activation of both MAPK/Erk1/2 and PI3K/Akt pathways. The regulation of astrocytic neurotrophic factor expression and secretion certainly contributes to MSC-mediated neuroprotection. In this context, related to our observation of differentially induced GDNF and CNTF-up-regulation in non-transgenic and SOD1G93A astrocytes, it is crucial to highlight and further characterize disease-related differences in basal and inducible growth factor expression levels. Last, our data confirm previously described functional modulation of astrocytes and microglia by MSC CM *in vitro*, again with distinct reactions of SOD1G93A glial cells. Future studies will need to more precisely define potential disease-related alterations in the reactivity to MSC CM and in the modulation of motor neuron-astrocyte-microglia crosstalk *in vitro* and *in vivo*. Our results support the potential of MSC to contribute to a more protective environment for degenerating neurons in ALS via regulation of growth factor, cytokine and chemokine secretion and therefore to be further evaluated as novel therapeutic approach for the treatment of ALS.
